# Ferroptosis and Ferro-Aging in Obesity: Bibliometric Mapping, Shared Mechanisms, and Translational Perspectives

**DOI:** 10.3390/metabo16090691

**Published:** 2026-09-18

**Authors:** Jiaxin Liu, Likun Zheng, Zhengri Cong, Zehao Liu, Chen Hu, Yuxin Liu, Chong Zhang, Mingjun Liu

**Affiliations:** 1 College of Acupuncture and Tuina, Changchun University of Chinese Medicine, Changchun 130117, China; ljxin1209@163.com (J.L.);; 2 College of Ophthalmology, Chengdu University of Chinese Medicine, Chengdu 610075, China; 3 College of Nursing, Wuyi University, Nanping 354300, China; 4 College of Acupuncture and Tuina, Chengdu University of Chinese Medicine, Chengdu 610072, China

**Keywords:** obesity, ferroptosis, ferro-aging, metabolites, ACSL4, lipid peroxidation, iron metabolism, cellular senescence, metabolic aging

## Abstract

**Highlights:**

**What are the main findings?**

- Bibliometric mapping of 564 publications identifies oxidative stress, lipid peroxidation, iron metabolism, and antioxidant defense as central themes in obesity-related ferroptosis research.
- Obesity may produce two distinct outcomes of iron–lipid peroxidation stress: acute ferroptotic cell death and proposed ferro-aging-like senescence under persistent sublethal stress.

**What are the implications of the main findings?**

- The proposed ferro-aging framework may help connect acute ferroptotic injury with chronic senescence-like metabolic tissue dysfunction, but it requires direct validation in obesity models.
- The broader ACSL4–iron–lipid peroxidation network and its parallel antioxidant defenses represent candidate intervention points requiring tissue-specific and clinical evaluation.

**Abstract:**

**Background**: Obesity is a chronic metabolic disease characterized by lipid overload, low-grade inflammation, oxidative stress, and progressive organ dysfunction. Ferroptosis, an iron-dependent form of regulated cell death driven by lipid peroxidation, has been increasingly implicated in obesity-related complications. However, an acute cell death-centered model does not fully explain the chronic senescence-like decline observed in metabolic tissues. The recently proposed concept of ferro-aging provides a potential framework for linking iron-dependent lipid peroxidation to cellular senescence and metabolic aging. **Methods**: In this review, we combined bibliometric mapping with mechanistic synthesis to characterize the evolving research landscape of ferroptosis in obesity and integrate the emerging concept of ferro-aging into this field. Bibliometric analysis was performed to screen qualified literature, and keyword co-occurrence and co-citation analyses were conducted to extract mainstream research hotspots. We further systematically summarized the pathological microenvironment constructed by obesity and the core molecular mediator connecting ferroptosis and ferro-aging, and proposed a pathological continuum hypothesis for obesity-mediated lipid peroxidation injury. **Results**: A total of 564 eligible publications were identified, including 455 original articles and 109 reviews, with a marked increase in publication output after 2020. Keyword and co-citation analyses highlighted oxidative stress, lipid peroxidation, iron metabolism, GPX4/Nrf2-mediated antioxidant defense, mitochondrial dysfunction, gut microbiota, NAFLD, and DCM as major research themes. Mechanistically, obesity creates a pro-ferroperoxidative microenvironment through iron dysregulation, polyunsaturated fatty acid enrichment, chronic inflammation, mitochondrial stress, and impaired antioxidant capacity. Within this context, ACSL4 is proposed as a candidate molecular hub that may link acute ferroptotic injury with chronic iron-lipid peroxidation-driven cellular senescence. We proposed a hypothesis-generating framework. In this framework, acute lipid peroxidation may promote ferroptotic cell death. Persistent sublethal iron-lipid peroxidation stress may contribute to ferro-aging-like senescence. **Conclusions**: To our knowledge, this is the first review to place ferro-aging within the obesity-ferroptosis framework. Targeting the ACSL4-lipid peroxidation axis and restoring antioxidant defense, particularly through Nrf2-GPX4-related pathways, may provide new translational opportunities for stratifying and managing obesity-related complications. However, clinical validation of ferro-aging biomarkers and intervention strategies remains urgently needed.

## 1. Introduction

### 1.1. Obesity and Ferroptosis

Obesity has become a global public health crisis, affecting more than one billion people worldwide [1]. Rather than merely reflecting excess adiposity, obesity is a complex chronic metabolic disease characterized by dyslipidemia, persistent low-grade inflammation, insulin resistance, and progressive metabolic dysfunction. These abnormalities substantially increase the risk of type 2 diabetes mellitus (T2DM), nonalcoholic fatty liver disease (NAFLD), cardiovascular disease, and certain malignancies, thereby impairing quality of life and imposing a considerable burden on healthcare systems [2,3].

Ferroptosis is an iron-dependent form of regulated cell death. It is characterized by iron overload and excessive accumulation of lipid peroxides [4]. Increasing evidence indicates that the metabolic disturbances associated with obesity—including lipid overload, polyunsaturated fatty acid enrichment, chronic inflammation, mitochondrial dysfunction, oxidative stress, and disrupted iron homeostasis—can increase cellular susceptibility to ferroptosis by promoting iron-dependent lipid peroxidation and weakening antioxidant defenses [5]. Ferroptosis-related injury has been implicated in multiple obesity-associated complications, including nonalcoholic steatohepatitis, pancreatic β-cell dysfunction, and metabolic cardiomyopathy [6,7,8]. These observations place ferroptosis at the intersection of lipid dysregulation, oxidative damage, and multi-organ metabolic injury. Nevertheless, ferroptosis primarily describes an acute cell-death outcome and may not fully account for the chronic, progressive, and senescence-like functional decline that develops in metabolic tissues during obesity.

### 1.2. Ferro-Aging: An Emerging Concept

In 2026, Liu et al. introduced the concept of “ferro-aging” and defined it as a chronic senescence-associated program triggered by iron accumulation and driven by lipid peroxidation, with acyl-CoA synthetase long-chain family member 4 (ACSL4) acting as a key effector [9]. In cellular and non-human primate aging models, this phenotype was associated with ACSL4 upregulation, persistent lipid peroxidation, and canonical markers of cellular senescence. Ferro-aging therefore differs conceptually from ferroptosis: the latter culminates in regulated cell death, whereas the former describes persistent sublethal stress and senescence-associated dysfunction. Importantly, current evidence is derived mainly from one foundational aging study, and ferro-aging has not been directly demonstrated in obesity. Its application to obesity should therefore be treated as a testable hypothesis rather than an established mechanism.

### 1.3. Scope and Objectives of This Review

This bibliometric-informed review maps the development of obesity-related ferroptosis research and integrates the resulting themes with a mechanistic synthesis of iron handling, lipid remodeling, antioxidant defense, and tissue-specific outcomes. On this basis, we propose a hypothesis-generating dual-outcome framework in which obesity creates a pro-ferroptotic metabolic environment characterized by iron dysregulation, polyunsaturated fatty acid enrichment, chronic inflammation, mitochondrial stress, and impaired lipid peroxide detoxification. Within this environment, acute and severe lipid peroxidation may promote ferroptotic cell death, whereas persistent sublethal iron-lipid peroxidation stress may contribute to senescence-associated ferro-aging-like changes. This proposed relationship has not been directly validated in obesity models and should not be interpreted as an established transition from ferroptosis to ferro-aging. Rather, it provides a testable framework for integrating acute tissue injury with chronic metabolic decline and for guiding future biomarker and intervention studies.

## 2. Bibliometric Landscape of Ferroptosis Research in Obesity

### 2.1. Data Sources and Analysis Strategies

We adopted a hierarchical multi-database retrieval strategy. WoSCC was used as the primary database for bibliometric mapping. Scopus was used to validate publication trends and the leading countries and institutions. PubMed was searched separately to identify randomized controlled trials related to ferroptosis and obesity. We used CiteSpace 6.4.R1 and the bibliometrix R 4.4.0 package to analyze publication trends, collaboration networks, co-citation patterns, keyword bursts, and thematic evolution in obesity-related ferroptosis research.

**Figure 1:**
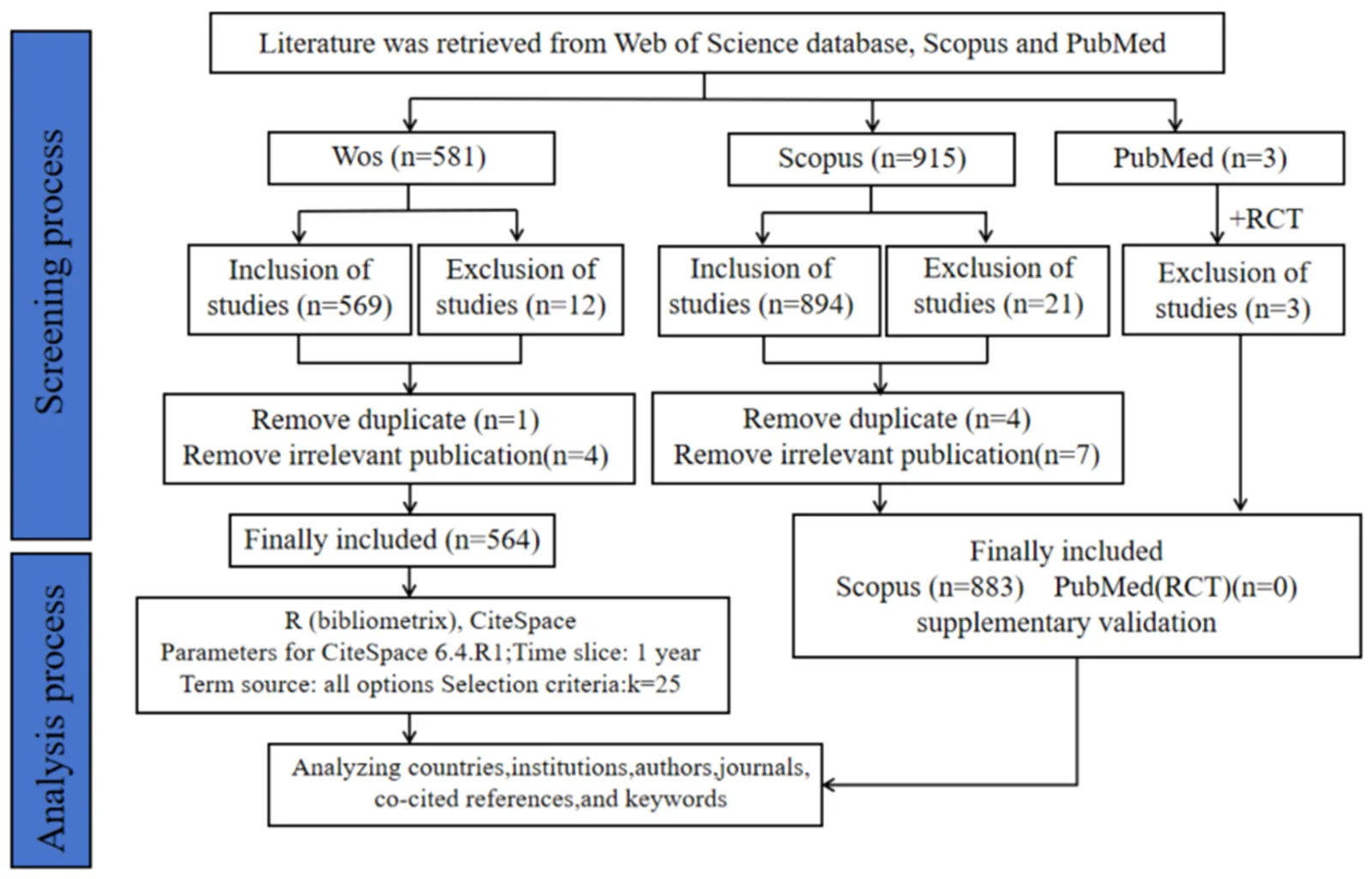
Retrieval Strategy and Literature Screening Process.

The retrieval period ranged from 1 January 2015, to 24 October 2025. Search terms were constructed using “ferroptosis”, “obesity”, and their synonyms. Two investigators (LJX and ZLK) independently screened the titles and abstracts, and disagreements were resolved through consultation with ZC. We included English-language original articles and reviews that addressed both ferroptosis-related mechanisms or functions and obesity-related human, animal, or cellular evidence. Conference abstracts, editorials, letters, book chapters, corrections, retracted records, and studies with low relevance were excluded. Figure 1 summarizes the retrieval, screening, and analysis process.

Ultimately, 564 eligible publications were included from the WoSCC, comprising 455 original articles and 109 reviews. CiteSpace and bibliometrix were used for co-occurrence, co-citation, burst-detection, and collaboration analyses. Cluster reliability was evaluated using modularity Q and silhouette S values. All reported clusters met the predefined reliability criteria. The complete software settings and analytical parameters are provided in the Supplementary Methods. Scopus data were analyzed using the “Analyze Search Results” function. No eligible randomized controlled trial was identified in PubMed.

### 2.2. Publication Trend and Global Contribution

**Figure 2:**
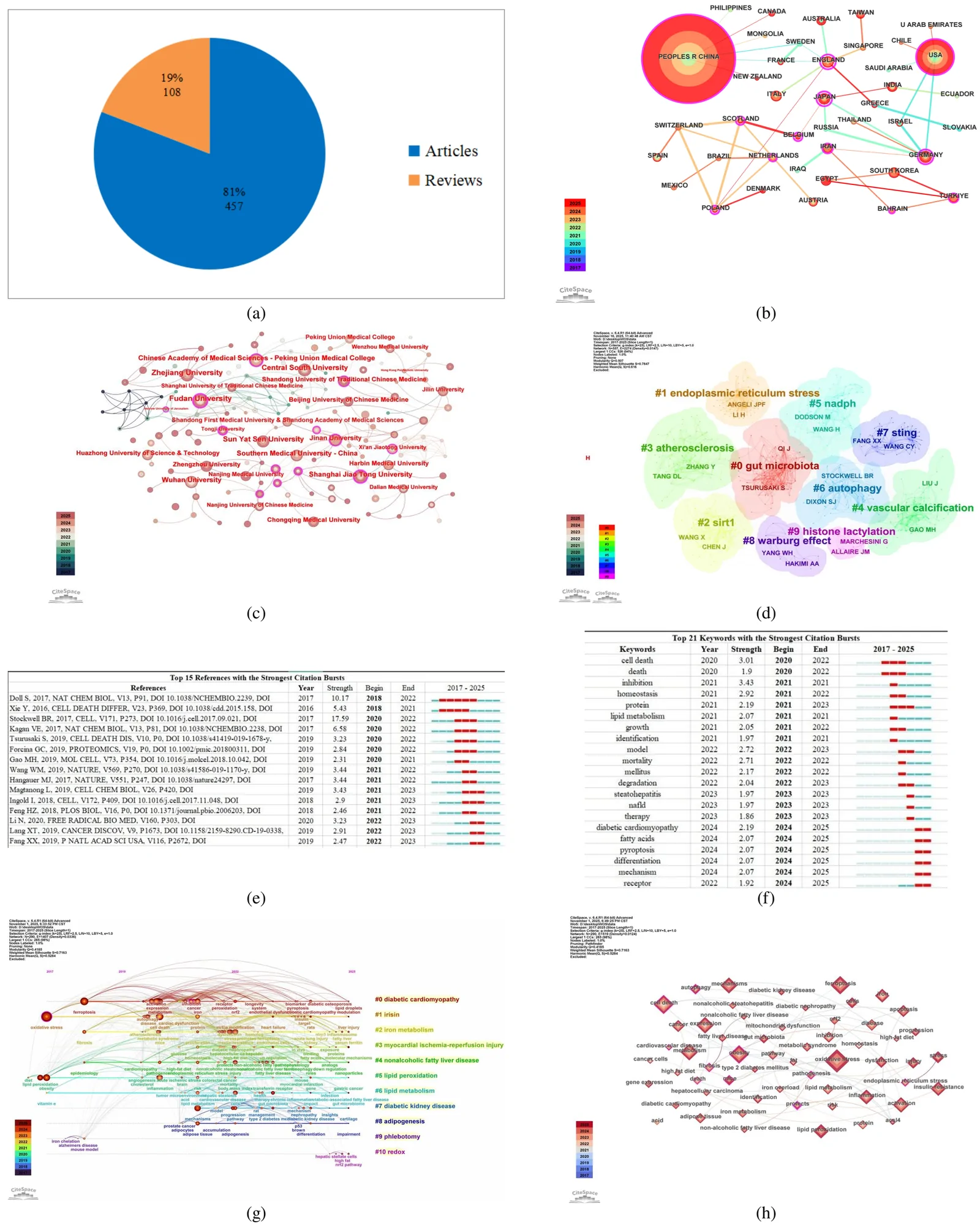
Bibliometric landscape and thematic signals informing the mechanistic synthesis of ferroptosis in obesity. (**a**) Annual publication trend; (**b**) Country/region collaboration network; (**c**) Institutional collaboration network; (**d**) Co–citation analysis network of authors; (**e**) References with the Strongest Citation Bursts, the cyan segments show the whole publication time range of each reference, while the red segments denote the period when the reference had a significant citation burst; (**f**) Keyword burst analysis; (**g**) Keyword timeline clustering; (**h**) Keyword co–occurrence network. Labels in panel (**e**) denote representative cited-reference nodes identified by CiteSpace and are displayed using the first author’s surname and publication year; they do not necessarily correspond to references formally cited in the manuscript.

Among the 564 publications ultimately included in this study, 455 were original research articles and 109 were review articles. The first publication addressing both ferroptosis and obesity appeared in 2017. Research output remained limited until 2019 but increased rapidly after 2020. A total of 438 publications appeared during the most recent three years of the retrieval period (Figure 2a). Scopus showed a comparable trend (Figure S1), indicating that this increase was not specific to a single database.

We further compared several growth models using RStudio 4.4.0 and the Bayesian Information Criterion (BIC). Growth-model comparisons supported a rapid increase in publication output. The power model showed the best overall fit, whereas the exponential model provided a closely comparable explanation of the trend. The complete BIC, AIC, goodness-of-fit, and curve-fitting results are provided in Table S2 and Figures S3–S5.

A total of 46 countries and regions worldwide have contributed to research on ferroptosis and obesity. Researchers from 46 countries and regions contributed to this field. China had the largest publication output, followed by the United States (Figure 2b). Japan, the United Kingdom, and Germany showed relatively high network centrality, indicating important roles in international collaboration. However, cross-regional collaboration between major Asian and Western research groups remained limited. The complete rankings and centrality values are presented in Table S3. Scopus confirmed the leading contributions of China and the United States (Figure S2).

A total of 199 institutions contributed to the WoSCC dataset. Fudan University showed the highest publication output and centrality in the institutional network (Figure 2c). Collaboration was concentrated among several leading Chinese universities, whereas broader cross-institutional networks remained underdeveloped. Detailed institutional rankings are provided in Table S3. Scopus produced a broadly comparable pattern, although the exact counts differed because of database coverage and institutional indexing (Table S1).

Institution-based keyword clustering identified gut microbiota, ubiquitination, and mitochondrial function as prominent research themes (Figure S6). These themes indicate that the field has expanded from conventional oxidative injury toward metabolic regulation, protein modification, and inter-organ communication.

### 2.3. Authors, Journals and Knowledge Foundation

A total of 496 researchers contributed to studies on ferroptosis and obesity. Wang Ying had the highest publication output, followed by Wang Qi, but the differences among the leading authors were small. The co-authorship network remained fragmented, with few stable large-scale research groups (Figure S7).

The co-cited author network identified Dixon SJ, Stockwell BR, Yang WS, Doll S, and Chen X as major contributors to the knowledge base of the field (Figure 2d). Dixon SJ had the highest co-citation frequency and centrality. These authors contributed foundational work on ferroptosis definition, lipid peroxidation, antioxidant defense, and cell-death regulation. The top author and co-cited authors rankings are provided in Table S4.

The journal dual-map overlay showed that knowledge flowed mainly from molecular biology and genetics toward medicine and related applied disciplines (Figure S8). This pattern indicates a close connection between basic ferroptosis research and metabolic disease studies.

In recent years, studies by Tsurusaki S , Li N , and Lang XT ) have shown notable citation bursts, as shown in Figure 2e. These studies linked ferroptosis with steatohepatitis, lipid peroxidation, and metabolic injury. Their emergence reflects the increasing focus on obesity-related organ damage rather than ferroptosis in cancer alone.

### 2.4. Keyword Bursts and Thematic Evolution

Figure 2f presents the top 21 keywords with the strongest citation bursts in the field of ferroptosis and obesity research. Among them, “cell death” showed the highest burst strength, followed by “inhibition” and “homeostasis”. More recent bursts included “diabetic cardiomyopathy (DCM)”, “fatty acids”, “differentiation”, and “receptors”. These changes indicate a shift from general cell-death research toward metabolic regulation and obesity-related organ complications.

Keyword timeline analysis identified 11 reliable clusters that centered on iron metabolism, lipid peroxidation, NAFLD, and diabetic cardiomyopathy (Figure 2g). In the co-occurrence network, “oxidative stress” had the highest frequency, followed by “ferroptosis,” “obesity,” and “cell death” (Figure 2h). The complete keyword frequencies and centrality values are provided in Table S4. The prominence of cell death-related terms indicates that the field remains strongly focused on acute injury. No established keyword directly represented chronic iron-dependent senescence or ferro-aging.

### 2.5. Summary of Bibliometrics

In conclusion, the bibliometric findings identify oxidative stress, lipid peroxidation, iron metabolism, antioxidant defense, NAFLD, and diabetic cardiomyopathy as the principal themes of obesity-related ferroptosis research. These findings guided the selection of the molecular pathways and tissue-specific contexts discussed in the following sections. However, the field remains dominated by acute cell-death terminology and preclinical disease models. Clinical translation is limited, and no eligible randomized controlled trial was identified in our targeted PubMed search. The ferro-aging concept was introduced after the bibliometric retrieval period and therefore did not appear in the analyzed literature. Nevertheless, the absence of terms addressing chronic iron-associated senescence reveals an important research gap. The following sections examine whether ferro-aging can provide a testable framework for investigating this gap while clearly distinguishing direct evidence from mechanistic inference.

## 3. Ferroptosis and Ferro-Aging: Mechanistic Overlap and Distinct Evidence Bases

### 3.1. Ferroptosis: Acute Cell Death Driven by Iron-Dependent Lipid Peroxidation

Ferroptosis is initiated when iron-catalyzed lipid peroxidation exceeds the capacity of cellular detoxification systems [10]. Intracellular labile iron generates hydroxyl radicals through the Fenton reaction, which then attack PUFA-containing membrane phospholipids. Meanwhile, ACSL4 catalyzes the conversion of long-chain PUFAs, such as arachidonic acid, into acyl-CoA esters and promotes their incorporation into membrane phospholipids. This process markedly increases membrane susceptibility to iron-dependent oxidative reactions [11,12].

In obesity, acute ferroptosis can occur in tissues under specific metabolic stress. Experimental studies indicate that hepatic ferroptosis can occur early during the progression of steatohepatitis and may promote subsequent liver inflammation [13]. High-glucose and palmitate exposure induced ferroptotic changes in pancreatic β-cell lines and primary mouse islets. Metformin attenuated these changes through regulation of the GPX4/ACSL4 axis [14]. In cardiomyocytes, obesity-associated lipid accumulation and oxidative damage can amplify ferroptotic signaling, promoting myocardial metabolic remodeling and contractile dysfunction [8].

### 3.2. Ferro-Aging: Definition, Experimental Basis, and Current Evidence Boundaries

In 2026, Liu et al. introduced the term “ferro-aging” in a study published in Cell Metabolism [9]. The authors described ferro-aging as a chronic senescence-associated process. Iron accumulation and lipid peroxidation characterized this process. The authors also identified ACSL4 as an important mediator. To assess the relationship between cellular senescence and iron homeostasis, the researchers measured iron levels across multiple human cell senescence models. In all models, senescent cells exhibited significant iron accumulation, upregulation of key lipid peroxidation enzymes including ACSL4, and elevated levels of reactive oxygen species (ROS) and malondialdehyde (MDA). To define this program at the molecular level, transcriptomic analyses were performed. Upregulated senescence genes were intersected with canonical factors of iron metabolism, lipid peroxidation, and antioxidant response, from which a ferro-aging score was calculated. The results showed that aged primates had significantly higher ferro-aging scores compared to younger primates. The study further supported that ACSL4 is a critical effector of iron-induced senescence: overexpression of ACSL4 promotes lipid peroxidation and cellular senescence, whereas ACSL4 knockdown or targeted intervention mitigates these processes.

Beyond the original primate study, ferro-aging-like programs have begun to be reported in other pathological contexts. For example, activation of a SIRT1-p53-related ferro-aging-like program was recently implicated in trophoblast dysfunction in preeclampsia. Although this evidence is not derived from obesity models, it supports the broader biological plausibility that iron-lipid peroxidation stress can converge with cellular senescence programs across disease contexts [15].

The differences between ferro-aging and ferroptosis are mainly reflected in their biological progression and cellular outcomes. Both processes involve iron-dependent lipid peroxidation and may involve overlapping molecular regulators, including ACSL4. However, they differ fundamentally in progression rate, injury intensity, and final outcome. Ferroptosis is an acute and high-intensity form of regulated cell death. It is usually triggered by extensive lipid peroxidation and leads to rapid membrane rupture and cell death [16]. In contrast, ferro-aging has been proposed as a chronic state associated with iron accumulation, lipid peroxidation, and cellular senescence. Current evidence does not establish whether persistent low-intensity lipid peroxidation directly causes this senescent state in obesity. Over time, this process contributes to systemic functional decline across multiple organs. From the perspective of signaling kinetics, ferroptosis represents the acute burst phase of this pathway, whereas ferro-aging corresponds to its chronic accumulation phase. This distinction extends the biological significance of iron-dependent lipid peroxidation beyond cell death to cellular senescence and systemic aging. Therefore, ferro-aging may provide a mechanistic bridge for explaining how obesity accelerates metabolic aging. Long-term nutrient excess and chronic inflammation may not directly induce cell death. Instead, they may impose persistent low-grade iron-lipid peroxidation stress, progressively driving cellular senescence and impairing tissue function.

**Figure 3:**
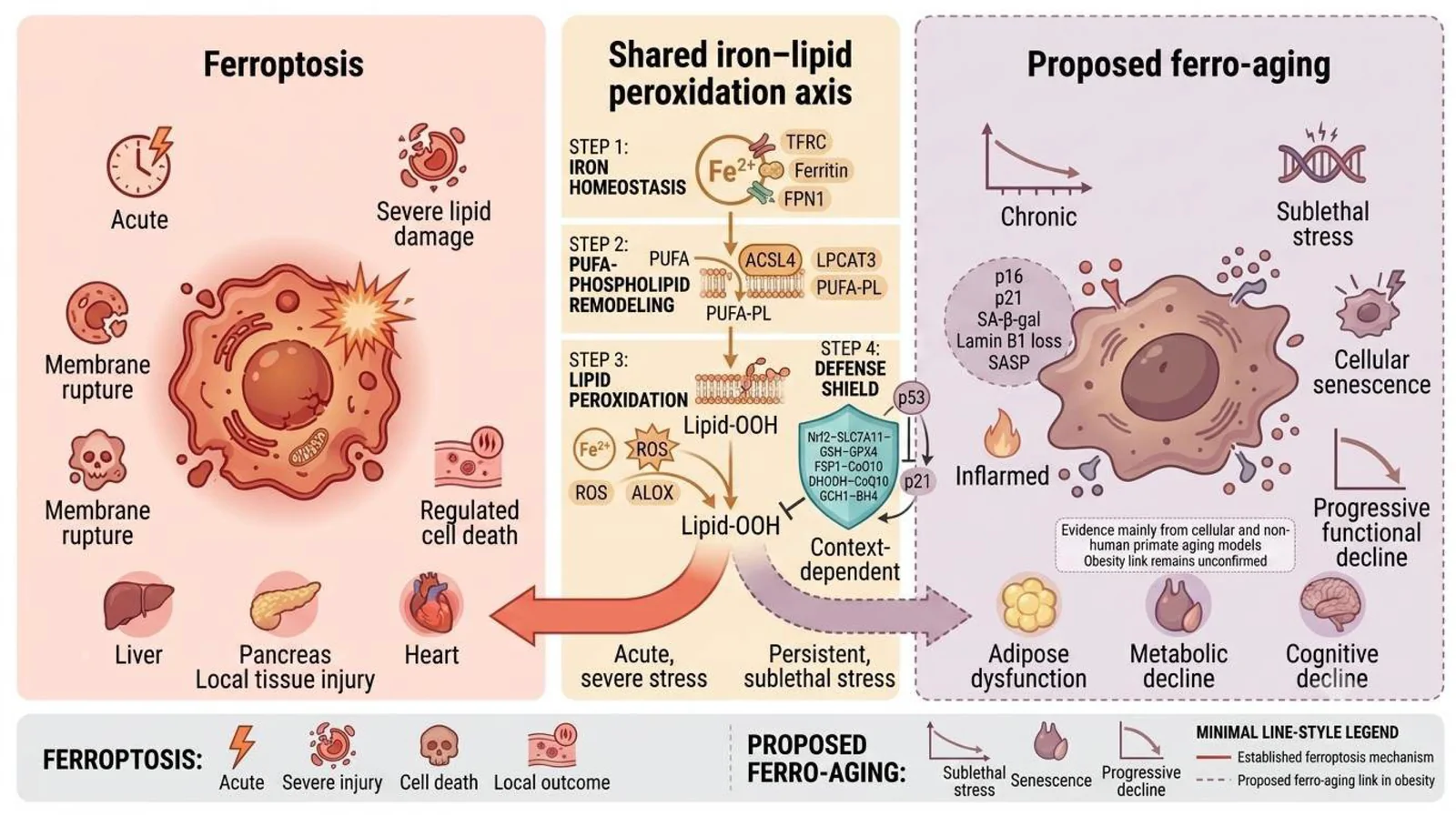
Proposed divergent outcomes of iron-dependent lipid peroxidation stress: ferroptosis and ferro-aging.

Importantly, ferro-aging should not be interpreted as a simple intermediate stage before ferroptotic death. Recent evidence suggests that senescent cells may acquire altered ferroptosis sensitivity through lysosomal dysfunction and iron compartmentalization. This observation reinforces the need to distinguish ferroptosis as a cell-death phenotype from ferro-aging as a chronic senescence-associated state [17]. Figure 3 presents the control models of ferroptosis and ferro-aging. Table 1 shows the key differences and overlaps between ferroptosis and ferro-aging. The available findings support ferro-aging as a distinct research concept. However, the current evidence base remains narrow. The original study requires independent replication in additional species, tissues, and disease models. Obesity-specific studies must also determine whether the same molecular program occurs during metabolic overload.

## 4. Obesity: A Metabolic Context for Ferroptosis and Potential Ferro-Aging

### 4.1. Obesity Creates a Pro-Ferroptotic Metabolic Microenvironment

Obesity is characterized by sustained nutrient excess, adipose tissue expansion, chronic low-grade inflammation, oxidative stress, and dysregulated lipid metabolism. Rather than acting through a single pathway, these abnormalities collectively create a metabolic environment that may increase susceptibility to iron-dependent lipid peroxidation. Increased availability of polyunsaturated fatty acids (PUFAs) expands the substrate pool for membrane phospholipid peroxidation, whereas ACSL4-mediated fatty acid activation and subsequent phospholipid remodeling may further increase the abundance of oxidation-prone PUFA-containing phospholipids [18,19]. In parallel, mitochondrial dysfunction, endoplasmic reticulum stress, and inflammatory signaling promote reactive oxygen species (ROS) production and place additional pressure on cellular antioxidant systems. When lipid peroxide generation exceeds the detoxification capacity of the SLC7A11-GSH-GPX4 and related defense systems, susceptible cells may undergo ferroptotic injury.

Iron homeostasis in obesity is more complex than generalized systemic iron overload. Elevated serum ferritin is frequently observed in individuals with obesity, but ferritin is also an acute-phase reactant and does not necessarily indicate expansion of the intracellular labile iron pool. Obesity-associated inflammation can increase hepcidin expression in adipose tissue and the liver, restrict ferroportin-mediated iron export, and promote iron sequestration in selected cellular compartments [20]. Therefore, circulating ferritin, serum iron, and tissue-accessible iron should not be treated as interchangeable indicators. Nevertheless, local iron retention may increase Fenton chemistry and lipid radical formation in metabolically stressed cells. This effect may be amplified by adipose tissue hypoxia, macrophage infiltration, mitochondrial dysfunction, and persistent inflammatory signaling.

These metabolic abnormalities provide a plausible biochemical context for ferroptosis, but increased iron levels, ROS, or lipid peroxidation alone are insufficient to establish ferroptotic cell death. Robust identification requires combined evidence of iron dependence, phospholipid peroxidation, cell death, and rescue by genetic or pharmacological inhibition of the ferroptosis pathway. Iron accumulation has also been shown to promote cellular senescence, senescence-associated secretory phenotype (SASP) activation, and fibrotic remodeling in non-obesity models [21,22]. These findings support the biological plausibility of a chronic iron-lipid peroxidation-senescence relationship. However, they do not demonstrate ferro-aging in obesity. Whether persistent iron-dependent lipid peroxidation induces a distinct ferro-aging program in obese metabolic tissues remains an open question requiring direct experimental validation.

### 4.2. Tissue- and Cell-Specific Outcomes in Obesity

The consequences of iron-dependent lipid peroxidation are unlikely to be uniform across metabolic organs. Tissue iron handling, membrane phospholipid composition, oxygen consumption, antioxidant capacity, and regenerative potential differ substantially among adipose tissue, liver, pancreatic islets, skeletal muscle, kidney, heart, and brain. Important differences may also exist among cell populations within the same organ. Consequently, lipid peroxidation may culminate in ferroptotic death in one cell type, induce sublethal stress in another, or contribute to adaptive metabolic remodeling under selected conditions. This heterogeneity argues against indiscriminate systemic inhibition of ferroptosis and supports the development of tissue-specific and temporally controlled interventions.

Adipose tissue provides a clear example of this context dependence. The capacity for ACSL4-mediated ferroptosis changes during brown-like adipogenic differentiation and is further modified by hypoxia [19]. Adipocyte-specific GPX4 deficiency increased adipose inflammation, glucose intolerance, and hepatic insulin resistance in mice. Notably, these abnormalities occurred without detectable adipocyte death, indicating that impaired lipid peroxide control can disrupt metabolic homeostasis independently of overt ferroptosis [23]. Conversely, Wang et al. showed that adipocyte-specific ACSL4 overexpression or ferritin heavy chain deletion activated ferroptotic signaling, promoted thermogenic programs, reduced lipid deposition, and protected mice against high-fat diet-induced obesity [24]. These findings indicate that ACSL4-associated lipid peroxidation in adipocytes is not uniformly detrimental. However, adaptive ferroptotic signaling under defined experimental conditions should not be equated with a general benefit of widespread adipocyte death. Evidence concerning adipose macrophages and stromal or progenitor cells remains limited, and the relative contributions of cell death, sublethal lipid peroxidation, and inflammatory remodeling require further investigation.

The liver, pancreatic islets, and heart show predominantly injury-associated patterns, although the strength and disease specificity of the available evidence differ. In a choline-deficient, ethionine-supplemented mouse model, hepatocyte ferroptosis preceded other forms of cell death and contributed to the initiation of steatohepatitis-associated inflammation [13]. Ferroptosis-related injury has also been reported in metabolic liver disease models, in which suppression of lipid peroxidation or restoration of antioxidant defense alleviated hepatic inflammation and injury [6,25,26,27]. However, findings obtained from nutrient-deficient or chemically induced steatohepatitis models should not be interpreted as direct evidence from obesity alone. Moreover, the respective contributions of hepatocytes, Kupffer cells, and hepatic stellate cells remain incompletely resolved and may differ across disease stages. Pancreatic β-cells have relatively limited antioxidant reserves and are vulnerable to glucolipotoxicity, mitochondrial stress, and lipid peroxide accumulation. Human islets exposed to erastin or RSL3 exhibited impaired viability, whereas ferrostatin-1 preserved islet viability and glucose-stimulated insulin secretion under ferroptosis-inducing conditions [28]. High-glucose and lipid-stress cell models also support β-cell susceptibility to ferroptotic injury [7]. Nevertheless, these experiments demonstrate ferroptotic susceptibility rather than proving that β-cell ferroptosis is a principal driver of obesity in vivo. Evidence concerning islet endothelial and immune cells remains insufficient. Ferroptosis has also been implicated in cardiomyocyte and endothelial injury in diabetic cardiomyopathy. Hyperglycemia-induced activation of the DNA-dependent protein kinase complex promoted endothelial ferroptosis and cardiac microvascular dysfunction in a type 2 diabetic cardiomyopathy model [29]. Although relevant to obesity-associated metabolic disease, evidence from diabetic cardiomyopathy should be distinguished from direct studies of obesity-induced cardiomyopathy and from ischemia-reperfusion or drug-induced cardiotoxicity models.

Emerging evidence also points to tissue-specific responses in skeletal muscle and the kidney, whereas direct evidence in the obesity-affected brain remains limited. In high-fat diet-fed mice, skeletal muscle cystathionine γ-lyase deficiency was associated with insulin resistance, ferroptosis-related changes, and muscle injury [30]. More recent work identified different patterns of GPX4, NCOA4, iron accumulation, and lipid peroxidation in red and white gastrocnemius muscles of obese mice, suggesting that ferroptotic susceptibility may differ even between muscle fiber types [31]. In the kidney, ferrostatin-1 attenuated high-fat diet-associated lipid peroxidation, inflammation, and renal injury in mice, providing preliminary evidence that ferroptosis contributes to obesity-related renal damage [32]. However, the respective roles of tubular epithelial, glomerular, endothelial, and immune cells have not been adequately resolved. Obesity-related neuroinflammation, blood-brain barrier dysfunction, and mitochondrial stress may increase ferroptotic vulnerability in the brain. However, most cell-specific evidence for neuronal, astrocytic, or microglial ferroptosis derives from neurodegenerative, ischemic, or aging models rather than obesity-specific studies. Accordingly, a direct obesity-brain ferro-aging pathway cannot currently be inferred.

**Figure 4:**
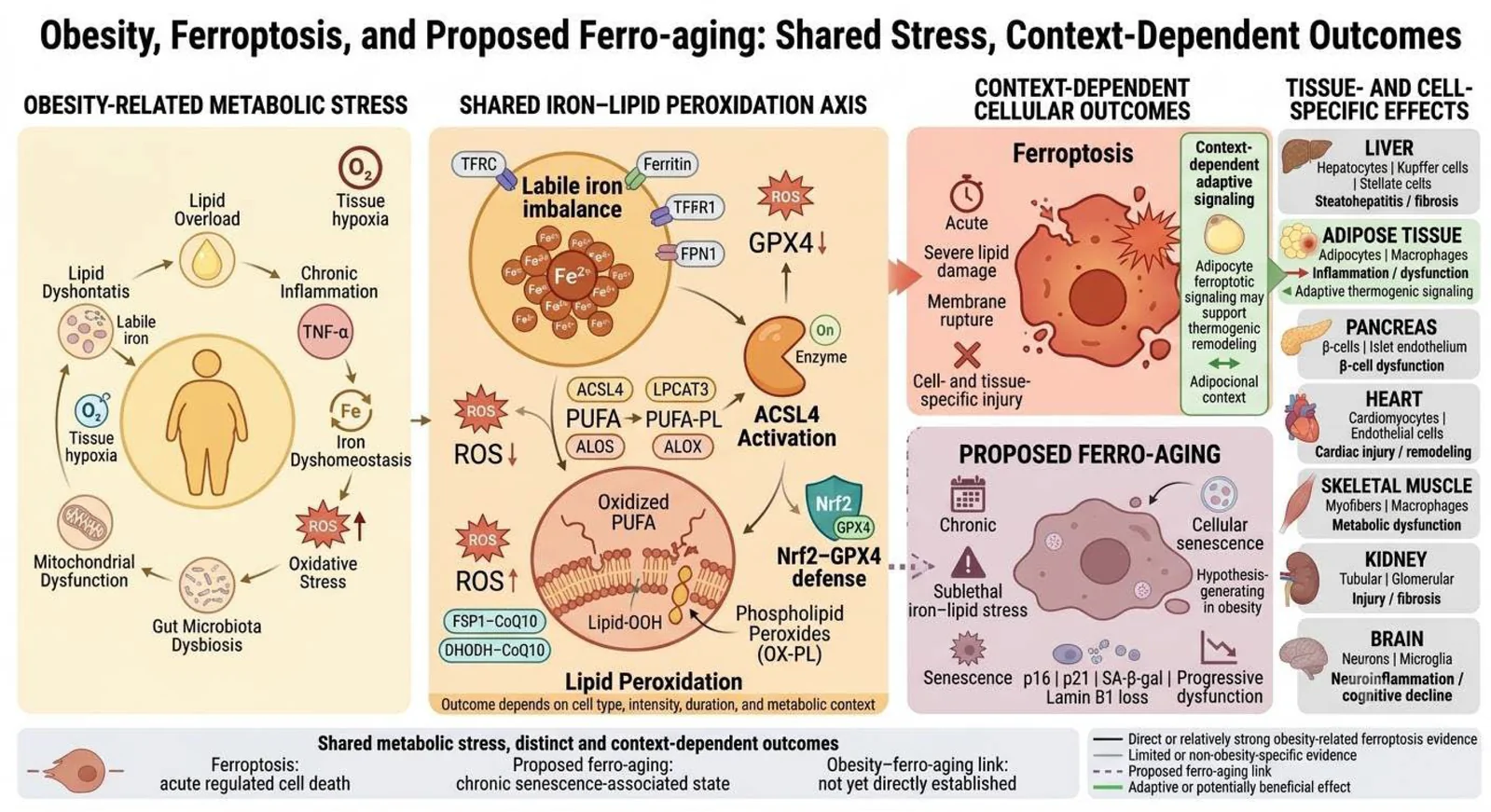
Obesity, Ferroptosis, and Ferro-aging: Shared Molecular Axis and Diverse Multi-Organ Complications.

Overall, obesity creates a shared background of nutrient overload, oxidative stress, altered iron handling, and increased availability of lipid peroxidation substrates, but the resulting cellular outcomes are determined by tissue context (Figure 4). Evidence for ferro-aging in these organs remains indirect. The proposed obesity-ferro-aging relationship is therefore based on convergent evidence involving iron dysregulation, persistent lipid peroxidation, cellular senescence, and fibrosis rather than on a directly demonstrated pathway. Future studies should combine tissue-specific genetic models with oxidized phospholipid profiling, iron measurements, ferroptosis rescue experiments, and canonical senescence markers to determine when iron-lipid peroxidation stress results in ferroptotic death, metabolic adaptation, or ferro-aging-like senescence. Table 2 presents the ferroptosis and potential ferro-aging mechanisms in obesity-associated complications.

### 4.3. Context-Dependent Effects of Ferroptotic Signaling in Obesity

The metabolic consequences of ferroptotic signaling may vary across tissues and cellular contexts. Ferroptosis is generally considered a form of regulated cell death that contributes to tissue injury when excessive lipid peroxidation overwhelms cellular defenses. However, recent evidence suggests that ferroptotic signaling may also exert adaptive metabolic effects under specific conditions. Adipose tissue provides an important example of this context-dependent effect. A recent study reported that ferroptotic signatures were reduced in adipose tissue from individuals and mice with obesity. The study further showed that a non-lethal dose of ferroptosis-inducing agents reduced lipid accumulation in primary adipocytes and limited adipose expansion in high-fat diet-fed mice. Adipocyte-specific Acsl4 overexpression or Fth deletion produced similar protective effects against obesity-associated adipose expansion and metabolic dysfunction. Mechanistically, ferroptotic signaling increased 5,15-dihydroxyeicosatetraenoic acid (5,15-DiHETE), promoted HIF1α degradation, and enhanced a c-Myc-PGC1β-dependent thermogenic program [24]. These findings suggest that ferroptotic signaling may have distinct metabolic consequences depending on its intensity and cellular context. In adipocytes, a controlled increase in lipid peroxidation-related signaling may promote thermogenic remodeling and limit excessive lipid storage. In contrast, excessive or sustained ferroptotic injury in metabolically important cells may impair tissue function. For example, ferroptosis in pancreatic β-cells has been associated with reduced cell survival and impaired insulin secretion, whereas ferroptosis inhibition can protect β-cell function under metabolic stress [34]. This context dependence has important implications for therapeutic strategies. Systemic inhibition of ferroptosis may protect vulnerable tissues from excessive lipid peroxidation, but it could also interfere with potentially adaptive ferroptotic signaling in selected metabolic tissues. Conversely, indiscriminate activation of ferroptosis may reduce adipose expansion but could damage insulin-producing or other metabolically important cells. Therefore, the therapeutic value of ferroptosis modulation may depend on tissue specificity, cellular context, and the intensity and duration of lipid peroxidation. This context dependence also provides an important boundary for the proposed ferroptosis-ferro-aging framework. Not all lipid peroxidation-associated signals should be interpreted as pathological ferro-aging. Adaptive signaling, reversible stress responses, and irreversible ferroptotic cell death may represent different biological outcomes. Whether persistent ferroptotic signaling in adipose tissue or other metabolic tissues contributes to ferro-aging remains unknown and requires direct experimental validation.

## 5. ACSL4 as a Candidate Convergence Point Within a Broader Iron–Lipid Peroxidation Network

### 5.1. ACSL4-Mediated PUFA Remodeling and Lipid Peroxidation

ACSL4 represents one of the most compelling molecular hubs linking these three fields. From a biochemical perspective, ACSL4 catalyzes the conversion of long-chain PUFAs, mainly arachidonic acid and adrenic acid, into acyl-CoA derivatives. These lipid intermediates are subsequently incorporated into membrane phospholipids. This process renders cellular membranes highly susceptible to iron-driven lipid peroxidation [35,36].

In canonical ferroptosis, ACSL4 is generally regarded as a key determinant of ferroptosis susceptibility. Its expression level is positively correlated with cellular sensitivity to ferroptosis inducers. Inhibition of ACSL4 attenuated tubular ferroptotic cell death and renal fibrosis in experimental kidney disease models [37]. In ferro-aging, ACSL4 was shown to drive chronic lipid peroxidation and cellular senescence. Liu et al. [9] showed that ACSL4 overexpression in young cells directly promotes lipid peroxidation and induces senescent phenotypes, whereas ACSL4 knockdown in senescent cells reverses these changes. This finding extends the role of ACSL4 from a “pro-death” factor to a “pro-senescence” regulator. The same team also conducted in vivo experiments. Mice fed a high-iron diet exhibited impaired cognitive and motor functions, together with increased markers of multi-organ aging. In contrast, liver-targeted CRISPR-Cas9-mediated knockout of ACSL4 significantly improved behavioral performance and reduced indicators of liver injury. Notably, ferro-aging-related genes showed the most pronounced age-associated changes in the liver, muscle, and adipose tissue of aged primates. This suggests that metabolically active tissues are particularly sensitive to this pathway. In addition to its intracellular role, ACSL4 may participate in intercellular propagation of senescence-associated lipid remodeling. Extracellular vesicle-packaged ACSL4 has been reported to induce lipid remodeling and senescent phenotypes in recipient hepatocytes, suggesting that ACSL4-mediated lipid peroxidation may contribute to tissue-level senescence signaling [38].

In the context of obesity, ACSL4 expression and function exhibit bidirectional regulation. On one hand, obesity can upregulate ACSL4 through multiple mechanisms, thereby increasing cellular susceptibility to iron-induced lipid peroxidation damage. On the other hand, Wang et al. [24] revealed a potential protective role of ACSL4-mediated ferroptotic signaling in adipose tissue. Adipocyte-specific overexpression of ACSL4 or knockout of ferritin heavy chain activated ferroptotic signaling, which induced thermogenic programs via hypoxia-inducible factor 1 alpha degradation and peroxisome proliferator-activated receptor gamma coactivator 1-beta pathway activation, thereby protecting mice from high-fat diet-induced adipose expansion and metabolic dysregulation. These findings suggest that the role of the “ACSL4/ferroptosis signal” in adipose tissue is context-dependent. In obesity, the balance between acute activation, which drive adaptive thermogenesis, and chronic sustained activation, which promotes senescence, ultimately determines the fate of metabolic tissues.

### 5.2. Other Regulatory Pathways of Ferroptosis and Lipid Peroxidation

Ferroptosis is regulated by multiple parallel pathways beyond the lipid remodeling pathway discussed above. These pathways control cysteine availability, glutathione metabolism, lipid peroxide clearance, mitochondrial redox balance, and radical-trapping antioxidant production. Their relative contribution varies across cell types and metabolic conditions. The SLC7A11-GSH-GPX4 axis represents a major intracellular defense system against lipid peroxidation. SLC7A11 imports cystine through the system Xc-transporter and supports cysteine availability for glutathione synthesis. GPX4 then uses GSH to reduce phospholipid hydroperoxides and maintain membrane integrity. Yang et al. demonstrated that GPX4 is an essential regulator of ferroptotic cell death, establishing the GSH-GPX4 system as a core ferroptosis defense pathway [39]. FSP1-CoQ10 provides another ferroptosis defense pathway that operates in parallel with GPX4. FSP1 reduces membrane-associated CoQ10 to ubiquinol using NAD(P)H. Ubiquinol acts as a lipid-soluble radical-trapping antioxidant and limits the propagation of lipid peroxidation. Two independent studies identified FSP1 as a glutathione-independent suppressor of ferroptosis [40,41]. These findings indicate that ferroptosis sensitivity depends on the balance between lipid peroxide generation and multiple antioxidant systems. Mitochondrial lipid peroxidation is also regulated by dihydroorotate dehydrogenase (DHODH). DHODH is located in the inner mitochondrial membrane and uses CoQ as an electron acceptor during pyrimidine biosynthesis. This reaction contributes to the maintenance of the reduced mitochondrial CoQ pool and limits mitochondrial lipid peroxidation. Mao et al. showed that DHODH provides a GPX4-independent ferroptosis defense mechanism, particularly in cells with low GPX4 expression [42]. The GCH1-BH4 pathway provides another GPX4-independent defense mechanism. GCH1 catalyzes the rate-limiting step in BH4 biosynthesis. BH4 can function as a radical-trapping antioxidant and can also influence lipid remodeling and CoQ10 metabolism. Kraft et al. showed that GCH1 and its downstream BH4/BH2 metabolites suppress ferroptosis by limiting the oxidation of phospholipids containing multiple PUFA chains [43]. These pathways may also be relevant to obesity-associated ferroptotic stress. Obesity is characterized by chronic oxidative stress, altered lipid metabolism, and impaired antioxidant capacity. These changes may alter the activity of GPX4-dependent and GPX4-independent defense systems. However, most mechanistic evidence for FSP1, DHODH, and GCH1 comes from non-obesity models. Their specific roles in obesity-associated ferroptosis and ferro-aging therefore require further validation.

## 6. The Nrf2–GPX4 Axis and Related Antioxidant Defense Networks

Within the regulatory networks of ferroptosis and ferro-aging, the Nrf2 signaling pathway and its downstream effector GPX4 constitute a shared endogenous defense barrier. GPX4 is currently the only known mammalian enzyme capable of reducing phospholipid hydroperoxides to phospholipid alcohols [39]. It has been extensively studied because of its central inhibitory role in ferroptosis. As a key regulator of cellular antioxidant responses, Nrf2 transcriptionally regulates multiple antioxidant and iron metabolism-related genes, including heme oxygenase-1 (HO-1), NAD(P)H quinone dehydrogenase 1 (NQO1), glutamate-cysteine ligase catalytic subunit (GCLC), and SLC7A11. Through these effects, Nrf2 provides upstream synergistic protection for GPX4-mediated defense. Yuan et al. [44] demonstrated that kaempferol enhances neuronal antioxidant capacity by activating the Nrf2/SLC7A11/GPX4 axis. It also suppresses the accumulation of lipid peroxidation in neurons. In addition, ML385, a specific inhibitor of this pathway, abolished the protective effects of kaempferol on neuronal antioxidant capacity, lipid peroxidation, and ferroptosis by inhibiting Nrf2. This reverse validation further supports the proposed mechanism [45,46].

Under obese conditions, the defensive function of the Nrf2-GPX4 axis is often impaired by chronic oxidative stress and nutrient excess. Experimental evidence from metabolic liver disease supports the involvement of the Nrf2-GPX4 defense system. In a mouse model of MASH, hepatocyte Nrf2 silencing increased ferroptosis-related changes and aggravated liver injury. Hinokitiol reduced these changes through hepatic Nrf2 activation [47]. Diosgenin also attenuated high-fat diet-induced hepatic steatosis, oxidative stress, and ferroptosis through Nrf2-related regulation [48]. Reduced GPX4 activity leads to depletion of GSH and triggers membrane phospholipid peroxidation [49]. At the same time, reduced nuclear translocation of Nrf2 decreases the transcription of downstream antioxidant genes. This further weakens cellular antioxidant reserves [50]. These findings suggest that restoring or enhancing the activity of the Nrf2-GPX4 axis may represent an effective strategy to concurrently inhibit ferroptosis and ferro-aging.

## 7. Therapeutic Implications and Translational Prospects

**Figure 5:**
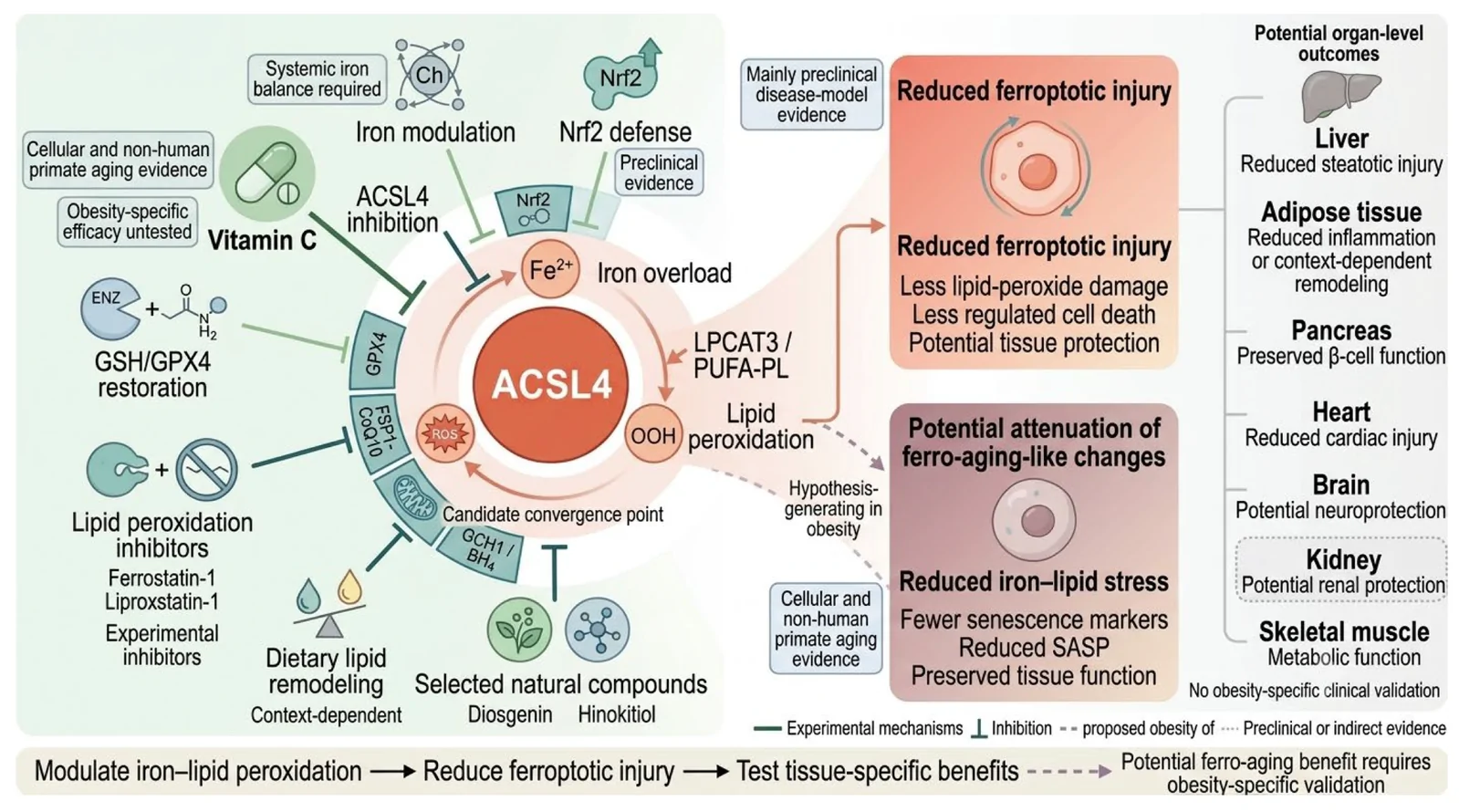
Therapeutic strategies targeting the ACSL4 axis to counteract ferroptosis and ferro-aging.

From a translational perspective, the ACSL4-lipid peroxidation axis provides a potential bridge between mechanism, biomarker development, and intervention. Clinically relevant translation will require the identification of obesity subgroups with high iron-oxidative burden, the development of non-invasive biomarkers reflecting lipid peroxidation and senescence, and the evaluation of interventions targeting ACSL4 activity, Nrf2-GPX4 defense, GSH metabolism, and lipid peroxide clearance. Vitamin C and other lipid peroxidation-modulating agents currently provide preclinical proof-of-concept, but their clinical utility in obesity-associated ferro-aging requires rigorous validation. Potential intervention strategies targeting this axis are summarized in Figure 5.

### 7.1. Biomarker Development

At present, clinical assessment mainly relies on tissue biopsy to detect lipid peroxidation end products, such as 4-hydroxynonenal (4-HNE) and MDA, or on transcriptomic sequencing to evaluate ferro-aging gene-set scores. A study of diabetic retinopathy reported alterations in iron- and oxidative stress-related indices that were interpreted in relation to ferroptotic injury [51]. However, these findings were obtained in a disease-specific setting. These circulating indices are not validated biomarkers of ferro-aging or obesity-associated ferroptosis. At present, no circulating biomarker panel has been validated for ferro-aging in obesity.

Because ferro-aging is defined as a senescence-associated process rather than a direct cell-death program, biomarker panels should include both iron-lipid peroxidation indicators and canonical senescence markers. These should include p16, p21, Lamin B1 loss, SASP factors, and tissue-specific transcriptomic or lipidomic signatures [52,53]. The application of multi-omics technologies, particularly lipidomics, single-cell transcriptomics, and spatial omics, will help establish specific ferro-aging signatures. Meanwhile, not all obese patients exhibit ferro-aging characteristics. Future research should identify obese subtypes with a high iron-oxidative burden and implement targeted interventions from the perspective of ferro-aging.

### 7.2. Vitamin C as as a Preclinical Proof-of-Concept

Based on the theoretical framework described above, identifying agents that target the ACSL4-lipid peroxidation pathway has become an important strategy for intervening in obesity-associated ferroptosis and ferro-aging. Liu Guanghui’s team screened approximately 100 ferroptosis-related small-molecule compounds and identified vitamin C as the most effective candidate. Mechanistic investigations revealed that vitamin C exerts a dual protective effect. The first mechanism involves direct targeting of ACSL4. Biotin-labeled vitamin C pull-down assays, LC-MS/MS analysis, and competition experiments confirmed a direct interaction between vitamin C and ACSL4. Molecular docking and enzymatic activity assays further identified the key binding sites through which vitamin C interacts with ACSL4. By inhibiting ACSL4-mediated PUFA-CoA production at its source, vitamin C reduces the availability of membrane phospholipid substrates for lipid peroxidation. The second mechanism involves direct activation of the Nrf2 pathway. Vitamin C promotes Nrf2 phosphorylation in a dose-dependent manner, thereby enhancing intrinsic cellular antioxidant defenses. In non-human primates, long-term vitamin C supplementation reduced ferro-aging signatures across multiple tissues. It also improved selected neurological and metabolic functions and lowered the biological age estimated by multi-omics aging clocks. Vitamin C provides an important mechanistic example, but its relevance to obesity requires careful interpretation. Future studies should test this mechanism in diet-induced and genetic obesity models. Clinical studies should also define the effective dose, tissue distribution, long-term safety, and relevant metabolic endpoints.

### 7.3. Restoring Nrf2-GPX4 Defense

Because both ferroptosis and ferro-aging involve lipid peroxide accumulation, strengthening detoxification pathways is another rational therapeutic strategy. Potential approaches include supporting GSH metabolism, preserving GPX4 function, activating Nrf2 signaling, and supplementing nutritional or pharmacological molecules with lipid peroxidation-inhibitory activity [54]. This strategy may be relevant to individuals with obesity who exhibit impaired antioxidant defense and an increased lipid peroxidation burden, but its clinical efficacy remains to be established.

### 7.4. Opportunities with Natural Products and Traditional Chinese Medicine

Bibliometric analyses and our research background suggest that natural products and traditional Chinese medicine (TCM) may serve as important resources for modulating the iron-lipid peroxidation axis. In preclinical NAFLD and MASH models, diosgenin and hinokitiol alleviated metabolic liver injury through mechanisms involving Nrf2 activation, GPX4-associated antioxidant defense, and suppression of lipid peroxidation and ferroptosis [27,47,48]. These findings provide more direct metabolic-disease relevance than evidence obtained from cancer, drug-induced cardiotoxicity, or hypertension models. Nevertheless, they do not establish efficacy against obesity-associated ferro-aging. Future studies should determine whether these compounds directly regulate ACSL4 activity, iron handling, oxidized phospholipid profiles, and canonical senescence markers in diet-induced or genetic obesity models. For compound TCM formulations, it is also worth exploring whether they act via multi-target synergistic regulation of iron homeostasis, lipid peroxidation, the SASP, and the gut microbiota to mitigate obesity-associated metabolic aging. Table 3 shows the potential therapeutic strategies targeting the obesity-ferroptosis-ferro-aging axis.

## 8. Challenges and Future Directions

### 8.1. Conceptual Boundaries

The primary challenge is to define concepts precisely. Ferroptosis and ferro-aging may share components of the iron-lipid peroxidation network. However, researchers should not treat the two processes as equivalent or sequential states. Ferroptosis represents a regulated form of cell death, whereas ferro-aging is a chronic program leading to cellular senescence and functional decline in tissues. Given the biochemical complexity and context dependence of ferroptosis, future studies should follow standardized recommendations for ferroptosis research and combine lipid peroxidation assays, cell-death readouts, iron measurements, and rescue experiments with ferroptosis inhibitors. This is particularly important when distinguishing acute ferroptosis from chronic ferro-aging [55].

### 8.2. Gaps in Clinical Translation

Our targeted database search did not identify randomized controlled trials that specifically evaluated the obesity-ferroptosis or obesity-ferro-aging framework. The available therapeutic evidence remains predominantly preclinical. No validated clinical biomarker currently identifies ferro-aging in patients with obesity. This indicates that the field remains largely at the preclinical stage. Before ACSL4, vitamin C, or other intervention strategies can be proposed for clinical application, rigorous mechanistic validation and clinically stratified studies are required. Ideally, future trials should enroll obesity subgroups with a high iron-oxidative burden. They should simultaneously assess body weight, insulin sensitivity, fatty liver status, inflammation, iron metabolism, lipid peroxidation, and senescence biomarkers, rather than relying on a single metabolic endpoint.

### 8.3. Priority Directions for Future Research

This study proposes several priority directions for future experiments. (1) In obesity models, ferroptosis markers should be measured alongside ferro-aging markers. (2) The ACSL4-dependent effects should be compared across metabolically critical organs, including liver, adipose tissue, pancreatic islets, heart, and brain. (3) Causal relationships should be validated using tissue-specific ACSL4 knockout, pharmacological inhibition, and nutritional interventions. (4) Integrative analyses of gut microbiota, dietary fatty acid composition, iron intake, and host lipidomics should be conducted to elucidate multi-organ iron-lipid communication networks.

## 9. Conclusions

To our knowledge, this review is the first to place the emerging concept of ferro-aging within the obesity-ferroptosis research framework. Current evidence supports an important role for iron dysregulation and lipid peroxidation in several obesity-related tissue injuries. Current evidence also supports a relationship between iron accumulation and cellular senescence in non-obesity models. However, no study has directly established ferro-aging in obesity. The proposed obesity-ferroptosis-ferro-aging relationship therefore represents a hypothesis-generating framework.

ACSL4 may serve as a candidate convergence point within this framework. Other antioxidant and lipid peroxide defense pathways may also modify the cellular outcome. Vitamin C provides a mechanistic proof-of-concept in aging models, but obesity-specific efficacy has not been demonstrated. Future studies should validate ferro-aging markers in obesity models. These studies should also distinguish ferroptotic cell death from sublethal lipid peroxidation and cellular senescence. Such evidence will determine whether this framework can support biomarker development or tissue-specific intervention.

## Figures and Tables

**Table 1:** Key differences and overlaps between ferroptosis and ferro-aging.

Feature	Ferroptosis	Ferro-Aging
Definition	Iron-dependent regulated cell death driven by lipid peroxide overload	Proposed chronic iron-lipid peroxidation program leading to cellular senescence
Time scale	Acute or subacute	Chronic and progressive
Core mechanism	Fe^2+^-ACSL4-PUFA-PL peroxidation; GPX4-GSH failure	Persistent ACSL4-mediated lipid peroxidation
Cellular outcome	Membrane damage and cell death	Senescence, SASP *, and functional decline *
Representative markers	ACSL4, GPX4 ↓, SLC7A11 ↓, lipid ROS, MDA, 4-HNE	ACSL4, MDA, ROS ↑, 4-HNE *, p16 *, p21 *, SA-β-gal *^,^ Lamin B1 ↓ *
Relevance to obesity	Local organ injury in liver, β-cells *, and heart *	Systemic metabolic aging **, fibrosis *, and tissue dysfunction **
Therapeutic focus	Ferroptosis inhibitors, GPX4-GSH restoration, Nrf2 activation	ACSL4 modulation, vitamin C, antioxidant defense, senescence control *

Notes: * Supported by non-obesity aging or senescence models. ** Hypothesized in the context of obesity; direct experimental evidence is currently unavailable.

**Table 2:** Tissue-specific evidence for ferroptosis and potential ferro-aging in obesity-associated metabolic dysfunction.

Tissue/Organ	Principal Cells	Main Ferroptosis-Related Evidence	Ferro-Aging Evidence	Interpretation
Adipose tissue	Adipocytes; macrophages; stromal cells	GPX4 loss promotes metabolic inflammation without overt adipocyte death [23]; ACSL4-associated signaling may enhance thermogenesis and mitigate obesity [19,24].	No direct evidence	Context-dependent; death and sublethal signaling should be distinguished
Liver	Hepatocytes; Kupffer cells; stellate cells	Hepatocyte ferroptosis contributes to steatohepatitis and metabolic liver injury [6,13,25,26,27,32].	Iron-associated senescence and fibrosis are supported mainly by non-obesity models [21,22].	Relatively strong preclinical ferroptosis evidence; ferro-aging remains indirect
Pancreatic islets	β-cells; endothelial and immune cells	Human islets and β-cell models are susceptible to ferroptotic injury [7,28].	No direct evidence	Direct susceptibility evidence, but limited obesity-specific causality
Heart	Cardiomyocytes; endothelial cells; fibroblasts	Cardiomyocyte and endothelial ferroptosis occurs in diabetic cardiomyopathy models [29,33].	No direct evidence	Diabetes-related evidence should not be equated with obesity cardiomyopathy
Skeletal muscle	Oxidative and glycolytic myofibers	Obesity produces muscle type-specific changes in iron, GPX4, NCOA4, and lipid peroxidation [30,31].	No direct evidence	Emerging tissue-specific evidence
Kidney and brain	Renal and neural cell populations	Fer-1 attenuates high-fat diet-associated renal injury [32]; obesity-specific brain evidence remains limited.	No direct evidence	Renal evidence is preliminary; brain ferro-aging remains speculative

Note: Direct evidence refers to findings obtained in obesity, high-fat diet, or closely related metabolic disease models. Ferro-aging associations derived from aging, fibrosis, or non-obesity senescence models are classified as indirect evidence.

**Table 3:** Potential therapeutic strategies targeting the obesity-ferroptosis-ferro-aging axis.

Target	Representative Strategy	Main Action	Evidence Level	Key Limitation
ACSL4	Vitamin C; ACSL4 inhibitors	Reduces PUFA-CoA formation and lipid peroxidation substrate supply	Mechanistic/preclinical/non-human primate	No obesity-specific clinical evidence
Vitamin C	Long-term supplementation or derivative development	Direct ACSL4 inhibition and Nrf2 activation	Non-human primate aging evidence	No obesity-specific efficacy evidence
GPX4-GSH axis	GSH support; GPX4-preserving strategies	Enhances lipid peroxide detoxification	In vitro/animal	No obesity-specific clinical evidence
Nrf2 pathway	Nrf2 activators; natural compounds	Upregulates antioxidant and iron-handling genes	In vitro/animal	Context-dependent effects; risk of nonspecific activation
Iron homeostasis	Iron chelation or hepcidin-ferroportin modulation	Lowers labile iron and Fenton-driven ROS	Mechanistic/preclinical	Excessive iron reduction may impair normal physiology
Lipid peroxidation	Ferrostatin-1, liproxstatin-1, vitamin E-like agents	Interrupts lipid radical chain reactions	Preclinical	Delivery, pharmacokinetics, and long-term safety remain unclear
Natural products/TCM	Diosgenin, hinokitiol, compound formulas	Multi-target regulation of Nrf2, GPX4, ACSL4, inflammation, and microbiota	In vitro/animal	Complex composition, quality control, and target validation challenges

Abbreviations: TCM, traditional Chinese medicine; PUFA-CoA, polyunsaturated fatty acyl-CoA; ROS, reactive oxygen species.

## Data Availability

No new data were created or analyzed in this study. Data sharing is not applicable to this article.

## Supplementary Materials

The following supporting information can be downloaded at: https://www.mdpi.com/article/10.3390/metabo16090691/s1.
