# Supplementary material for: Ferroptosis and Ferro-Aging in Obesity: Bibliometric Mapping, Shared Mechanisms, and Translational Perspectives
Source: Metabolites. 2026 Sep 18;16(9):691. doi: 10.3390/metabo16090691 (PMC13608923; doi:10.3390/metabo16090691)
Supplement: Supplementary file 1 [file metabolites-16-00691-s001.zip › metabolites-4470655-supplementary.pdf]

Supplementary Methods.

1. Literature Search Strategy

The retrieval period ranged from January 1, 2015, to October 24, 2025. Search terms were constructed using “ferroptosis”, “obesity”, and their synonyms. The search strategy employed was: TS1 = ("Obesity" OR "Adiposity" OR "Metabolic Syndrome" OR "Dysmetabolic Syndrome X" OR "Visceral Obesity" OR "Abdominal Obesity" OR "Fat"); TS2 = ("Ferroptosis"); TS = TS1 AND TS2.

WoSCC records were exported as “Full Record and Cited References”， whereas Scopus records were obtained in comma-separated values (CSV) format with complete bibliographic information.

Two investigators (LJX and ZLK) independently screened the retrieved literature. Disagreements were resolved through consultation with ZC.

The reviewers examined article titles, abstracts, and reference lists. All included studies were further cross-checked for eligibility. The inclusion criteria were as follows. (1) Eligible studies had to be published as original research articles or review articles. (2) The study had to simultaneously address two major themes: 1) ferroptosis-related research, including its biological levels, metabolic mechanisms, or functional roles; and 2) obesity-related human studies, animal experiments, or cellular mechanistic investigations. (3) Only studies published in English were included. (4) The publication period was restricted to studies published between January 1, 2015 and October 24, 2025.

The exclusion criteria were as follows. (1) Non-research or non-review publications were excluded, including conference abstracts, editorials, letters to the editor, book chapters, corrections, retracted publications, and early-access articles. (2) Studies with low relevance to the research topic were excluded.

2. Citespace Analysis Strategy

A total of 564 WoSCC records were included, comprising 455 original articles and 109 reviews. After data export, duplicate records were removed using CiteSpace 6.4.R1. Co-occurrence analysis, co-citation analysis, burst detection, and collaboration network visualization were performed using CiteSpace and bibliometrix R package. Figure organization and graphical refinement were conducted using Microsoft Excel 2019 and Adobe Illustrator 16.0. CiteSpace parameters were set as follows: the time slice was set to 1 year, the pruning method was Pathfinder, and the selection criterion was based on the g-index k = 25. Additional parameters included LRF = 2.5, L/N = 10, LBY = 5, and e = 1.0. Cluster quality was evaluated using the modularity Q value and the silhouette S value. A Q value greater than 0.3 generally indicates a significant clustering structure, whereas an S value greater than 0.7 indicates high internal consistency within clusters. All clustering results in this study

met these reliability criteria. Scopus data were analyzed directly using its built-in "Analyze Search Results" function. The RCT records retrieved from PubMed were initially planned for analysis using bibliometrix R package. However, no eligible RCTs were identified, and therefore no subsequent statistical analysis was conducted.

Supplementary Figures

Supplementary Figure S1. Annual publication trend in research on ferroptosis and obesity based on Scopus data, 2015–2025.

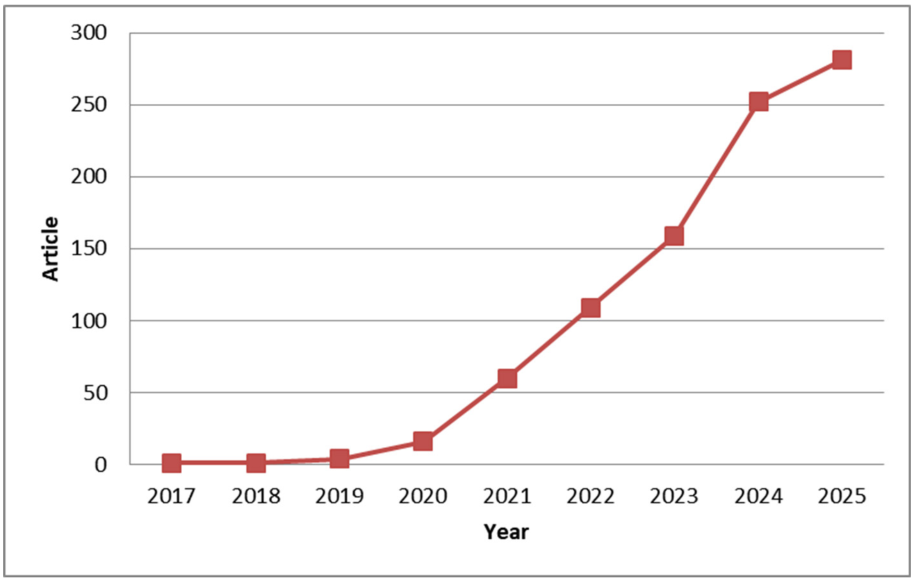

Supplementary Figure S2. Top 10 countries/regions contributing to research on ferroptosis and obesity based on Scopus data, 2015－2025.

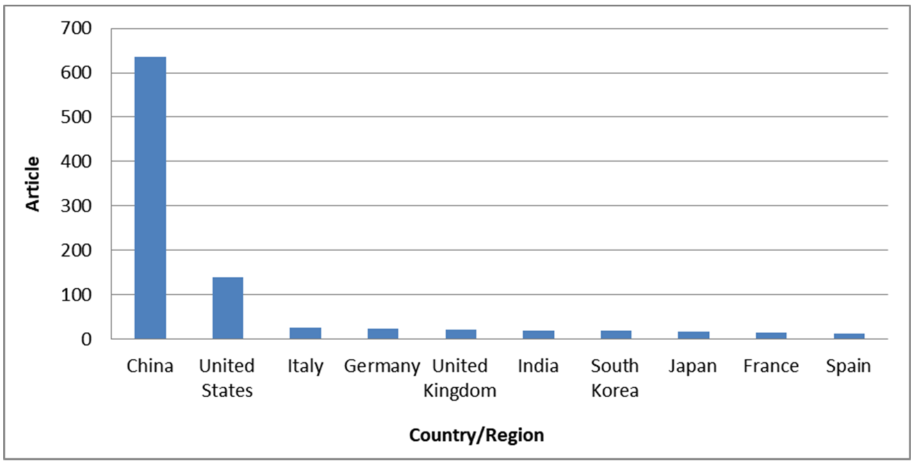

Supplementary Figure S3. Comparison of ΔBIC values among candidate models. Smaller ΔBIC values indicate better model fit to annual publication volume data.

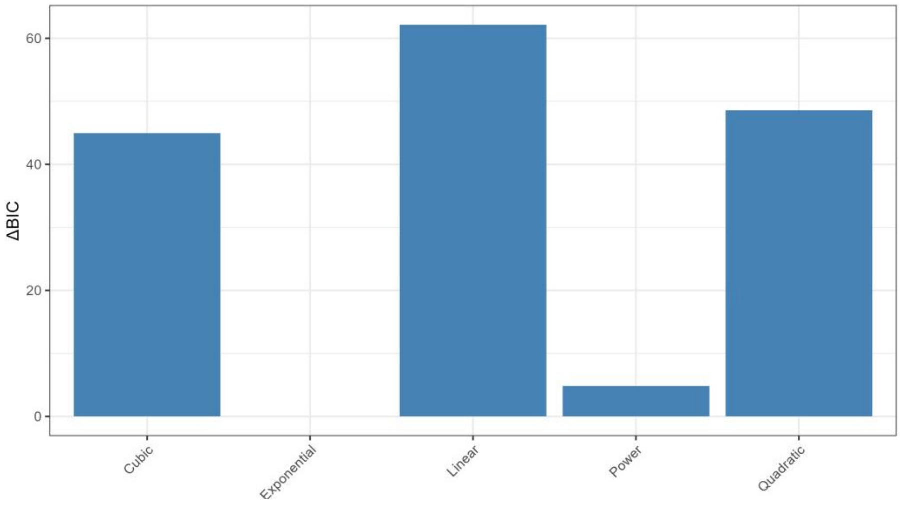

Supplementary Figure S4. Index model fitting curve for annual publication volume of ferroptosis research on obesity.

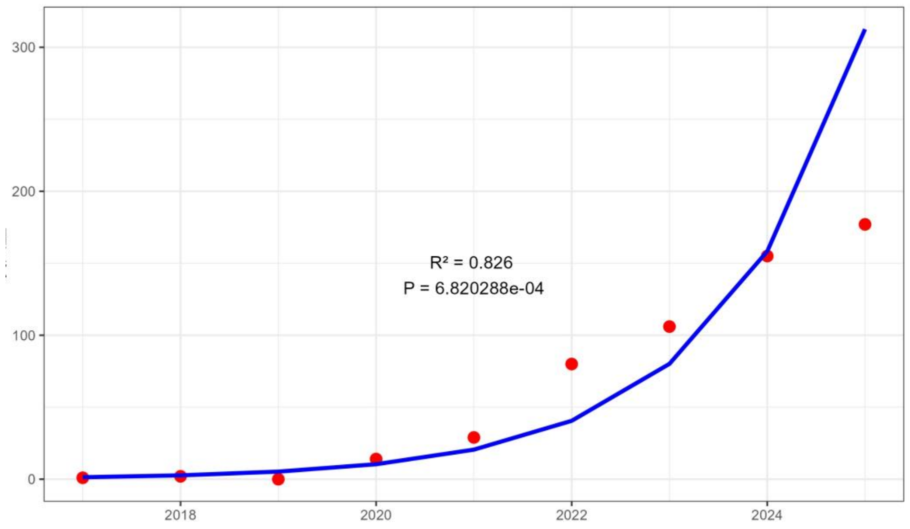

Supplementary Figure S5. Power model fitting curve for annual publication volume of ferroptosis research on obesity.

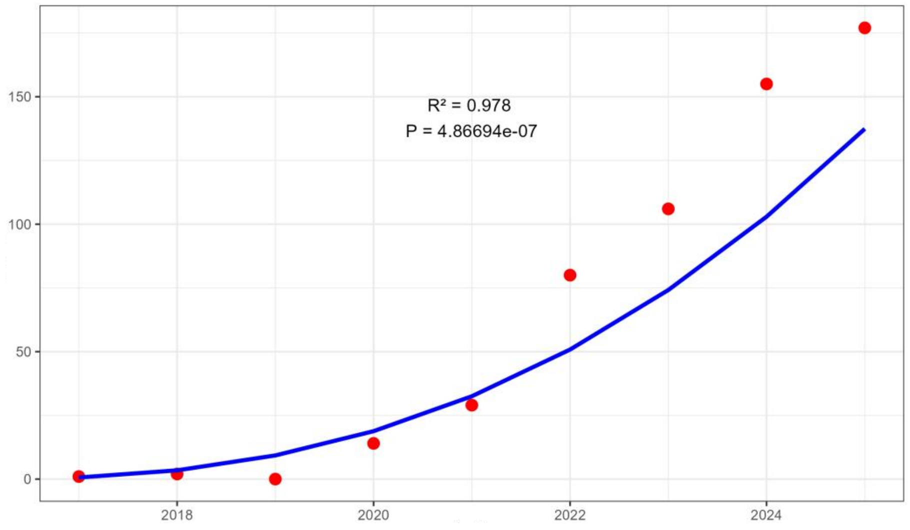

Supplementary Figure S6. Keyword Clustering Network.

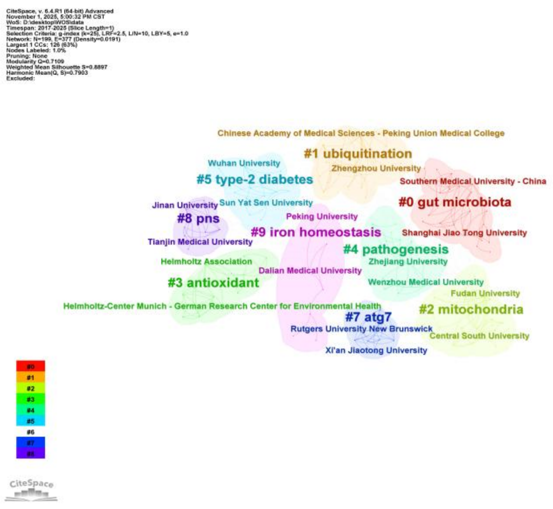

Supplementary Figure S7. Author Collaboration Network.

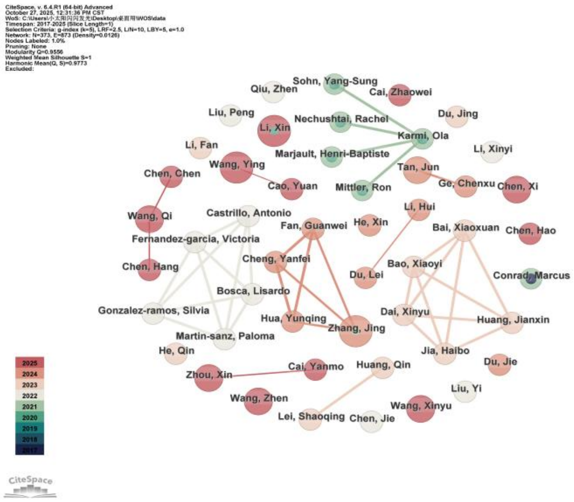

Supplementary Figure S8. Journal dual-map overlay.

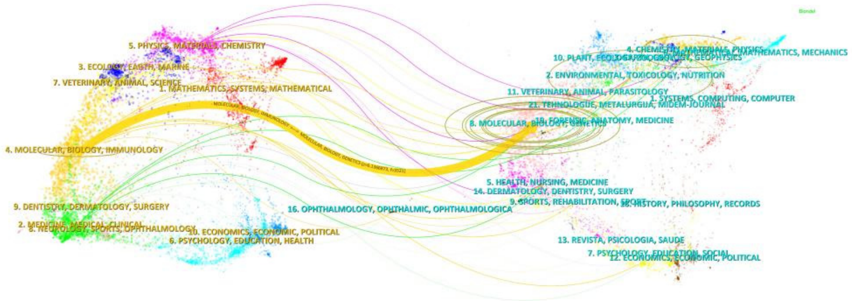

Supplementary Tables

Supplementary Table S1. Top 10 institutions contributing to publications on ferroptosis in obesity research in Scopus(2015–2025).

Institution	Article
Ministry of Education of the People's Republic of China	64
Fudan University	28
Sun Yat-Sen University	24

Chinese Academy of Medical Sciences & Peking Union Medical College	23
Southern Medical University	19
Central South University	19
Shanghai Jiao Tong University School of Medicine	19
Beijing University of Chinese Medicine	18
Zhejiang University School of Medicine	17
First Affiliated Hospital of Zhengzhou University	17

Supplementary Table S2. Δ BIC and AIC values for each model.

ΔBIC values of each model				
Power	Exponential	Linear	Quadratic	Cubic
4.818027	0.000000	62.159506	48.602751	44.952164
**ΔAIC values of each model**				
Power	Exponential	Linear	Quadratic	Cubic
29.24903	24.43100	86.59051	72.83653	68.98872

Supplementary Table S3. Top countries/regions and institutions.

Rank	Country	Centrality	Year	Publications
1	PEOPLES R CHINA	0.3	2020	449
2	USA	0.23	2018	75
3	GERMANY	0.76	2017	13
4	ENGLAND	0.82	2020	11
5	ITALY	0	2020	10
6	JAPAN	0.83	2021	10
7	SOUTH KOREA	0	2022	7
8	INDIA	0.08	2022	7
9	EGYPT	0	2024	6
10	TAIWAN	0	2023	5
11	SPAIN	0	2022	5
12	AUSTRALIA	0	2021	5
13	TURKIYE	0.15	2024	5
14	IRAN	0.36	2021	5

Rank	Institution	Centrality	Year	Publications
1	Fudan University	0.31	2020	19
2	Zhejiang University	0.07	2021	18
3	Sun Yat Sen University	0.08	2022	17
4	Central South University	0.07	2020	16
5	Wuhan University	0.06	2022	15
6	Southern Medical University - China	0.09	2022	15
7	Chinese Academy of Medical Sciences - Peking Union Medical College	0.12	2021	15
8	Shanghai Jiao Tong University	0.3	2020	15
9	Jinan University	0.15	2023	13
10	Chongqing Medical University	0	2023	12
11	Zhengzhou University	0.07	2022	12
12	Huazhong University of Science & Technology	0.01	2023	11
13	Harbin Medical University	0.01	2023	11
14	Beijing University of Chinese Medicine	0.02	2023	11
15	Shandong University of Traditional Chinese Medicine	0.15	2022	11

Supplementary Table S4. Top authors and co-cited authors.

	Rank	Name	Centrality	Year	Publications
Author	1	Wang, Ying	0	2023	6
	2	Wang, Qi	0	2023	5
	3	Li, Hui	0	2024	4
	4	Zhang, Jing	0	2021	4
	5	Li, Xin	0	2020	4
	6	Zhang, Yi	0	2024	4
	7	Qiu, Zhen	0	2022	4
	8	Zhou, Xin	0	2025	3
	9	Tan, Jun	0	2024	3
	10	Yang, Guang	0	2020	3
Co-Author	1	DIXON SJ	0.35	2017	242
	2	STOCKWELL BR	0.08	2020	158
	3	YANG WS	0.12	2020	130

4	CHEN X	0.15	2021	116
5	LI J	0.08	2020	107
6	WANG Y	0.09	2022	99
7	DOLL S	0.17	2020	98
8	JIANG XJ	0.09	2021	97
9	FANG XX	0.07	2020	77
10	GAO MH	0.12	2020	75
11	TANG DL	0.01	2022	66

Supplementary Table S5. Top high-frequency keywords.

Rank	Keyword	Centrality	Year	Publications
1	oxidative stress	0.14	2017	127
2	ferroptosis	0.05	2018	76
3	obesity	0.1	2017	75
4	cell death	0.08	2020	75
5	metabolism	0.04	2020	68
6	mechanisms	0.04	2021	59
7	expression	0.02	2020	59
8	activation	0.02	2020	55
9	inflammation	0.03	2020	54
10	lipid peroxidation	0.02	2017	50
11	iron	0.02	2021	43
12	insulin resistance	0.02	2022	43
13	apoptosis	0.06	2020	39
14	cancer	0.04	2021	35
15	stress	0.03	2022	32
16	dysfunction	0.01	2022	30
17	disease	0.02	2020	30
18	inhibition	0.1	2021	29
19	cells	0.01	2022	29
20	lipid metabolism	0.09	2021	28
